# Prognostic value of fibrinogen to albumin ratios among critically ill patients with acute kidney injury

**DOI:** 10.1007/s11739-021-02898-3

**Published:** 2021-11-30

**Authors:** Wenkai Xia, Chenyu Li, Xiajuan Yao, Yan Chen, Yaoquan Zhang, Hong Hu

**Affiliations:** 1 Department of Nephrology, The Affiliated Jiangyin Hospital of Southeast University Medical College, 3 Yinrui Road, Jiangsu, 214400 Jiangyin China; 2 Nephrologisches Zentrum, Medizinische Klinik und Poliklinik IV, Klinikum der Universität München, Ludwig-Maximilians-University Munich, Munich, Germany

**Keywords:** Fibrinogen, Albumin, Acute kidney injury, Prognosis

## Abstract

Fibrinogen to albumin ratios (FAR) have shown to be a promising prognostic factor for improving the predictive accuracy in various diseases. This study explores FAR's prognostic significance in critically ill patients with acute kidney injury (AKI). All clinical data were extracted from the Multiparameter Intelligent Monitoring in Intensive Care Database III version 1.4. All patients were divided into four groups based on FAR quartiles. The primary endpoint was in-hospital mortality. A generalized additive model was applied to explore a nonlinear association between FAR and in-hospital mortality. The Cox proportional hazards models were used to determine the association between FAR and in-hospital mortality. A total of 5001 eligible subjects were enrolled. Multivariate analysis demonstrated that higher FAR was an independent predictor of in-hospital mortality after adjusting for potential confounders (HR, 95% CI 1.23, 1.03–1.48, *P* = 0.025). A nonlinear relationship between FAR and in-hospital mortality was observed. FAR may serve as a potential prognostic biomarker in critically patients with AKI and higher FAR was associated with increased risk of in-hospital mortality among these patients.

## Introduction

Acute kidney injury (AKI) is a prevalent complication in critically ill patients, which results in long length of intensive care unit (ICU) stay, increased morbidity and mortality, and high risk of chronic kidney disease (CKD) and end-stage renal disease (ESRD) [1, 2]. Emerging evidence has demonstrated that early screening and managing the risk factors associated with AKI prevent in-hospital mortality of critical patients [3, 4]. Therefore, it is necessary to identify economic and reliable clinical indicators that can outperform or add significant value to the existing conventional score system for ICU patients with AKI.

The mechanisms underlying AKI are complicated and multifactorial, it is well recognized that inflammation is involved in the initiation and progression of AKI [5, 6]. Recent studies suggested that systemic inflammatory biomarkers can be utilized as potential predictors for AKI patients [7–9]. As a novel inflammation-based indicator, fibrinogen to albumin ratio (FAR) has gained prognostic value in various cancers [10–12] and myocardial infarction [13]. Additionally, several studies recently reported that FAR levels are associated with contrast-induced nephropathy and the occurrence of post-contrast acute kidney injury [14, 15]. However, data on the association of the FAR with prognosis of AKI patients are limited. The purpose of this study is to investigate the prognostic value of FAR in predicting in-hospital mortality in critically ill patients with AKI.

## Methods

### Data source

Our study was based on a publicly available Multiparameter Intelligent Monitoring in Intensive Care III version 1.4 (MIMIC-III v1.4). The database includes more than 40,000 critically ill patients admitted to Beth Israel Deaconess Medical Center (Boston, MA, USA) from 2001 to 2012 [16]. Access to the database for research was approved by the Institutional Review Boards of the Massachusetts Institute of Technology (Cambridge, MA, USA) and the Beth Israel Deaconess Medical Center. Given that all data were deidentified, informed consent was waived by the ethical committee of the Beth Israel Deaconess Medical Center. All methods were performed in accordance with the relevant guidelines and regulations.

### Population selection criteria

According to the Kidney Disease Improving Global Outcomes (KDIGO) classification, adult patients (≥ 18 years) with AKI who had been hospitalized in the ICU at first admission for more than 2 days were included. Patients who met the following criteria were excluded: (1) no albumin or fibrinogen measured during the ICU stay; (2) missing > 5% individual data.

### Data extraction

Structured query language (SQL) with PostgreSQL (version 9.6) was used to perform data extraction from MIMIC-III. The comorbidities included atrial fibrillation (AFIB), coronary artery disease (CAD), congestive heart failure (CHF), renal disease, chronic liver diseases, diabetes, chronic obstructive pulmonary disease (COPD), pneumonia, acute respiratory distress syndrome (ARDS) and stroke. The laboratory parameters were also extracted, including white blood cell (WBC), hematocrit, hemoglobin, platelet, anion gap (AG), prothrombin time (PT), activated partial thromboplastin time (APTT), albumin, fibrinogen, blood urea nitrogen (BUN), creatinine, glucose, bicarbonate, chloride, sodium, potassium and total bilirubin. The FAR was defined as the ratio of the fibrinogen level to the albumin level. Furthermore, Sequential Organ Failure Assessment (SOFA) scores and Simplified Acute Physiology Score (SAPS) II were calculated as described in previous studies [17, 18]. Only the data for the patient’s first ICU admission was used for assessment and baseline data was extracted within 24 h after ICU admission. The primary endpoint was hospital mortality.

### Statistical analysis

Baseline characteristics of all patients were stratified by FAR quartiles. Continuous variables were presented as mean ± standard deviation (SD) and categorical data were expressed as number or percentage. Chi-square, one-way ANOVA or Kruskal–Wallis tests were used to determine the significance difference between groups. The Cox proportional hazards models were constructed to examine the relationship between FAR levels and in-hospital mortality, and results were presented as hazard ratios (HRs) with 95% confidence intervals (CIs). We also used a generalized additive model (GAM) to identify the nonlinear association between FAR and in-hospital mortality. Three multivariate models were constructed on the basis of FAR group inclusion according to quartiles. The second quartile was treated as the reference group. Subgroup analysis was performed to evaluate the association between FAR and in-hospital mortality, including age, gender, laboratory parameters, comorbidities, SOFA and SAPS II score. Receiver-operating characteristic (ROC) curve was applied to evaluate predictive ability of FAR and existing scoring system (SOFA score and SAPS II score) on in-hospital mortality. All statistical analyses were performed using SPSS 20.0 software (SPSS Inc., IBM, USA) and R software version 3.4.2 (Institute for Statistics and Mathematics, Vienna, Austria). A two-tailed value < 0.05 was considered statistically significant.

## Results

### Subject characteristics

A total of 5001 eligible subjects were enrolled in our study. Characteristics of these patients by quartiles of FAR were summarized in Table 1. There were 2066 women and 2935 men with a mean age of 63 ± 16 years. Patients in top quartile of FAR were more likely to be elderly with a history of AFIB, CHF, renal disease, pneumonia, ARDS, malignancy and diabetes, as well as higher values of WBC, creatine, BUN, anion gab, platelet, SOFA score, SAPSII score, ICU LOS, and mortality.

**Table 1:** Baseline characteristics of participants according to FAR

Characteristics	FAR				*P* value
	Q1(< 0.059)	Q2(0.059- < 0.093)	Q3(0.093- < 0.157)	Q4(≥ 0.157)	
Ages, years	60 ± 17	63 ± 16	65 ± 16	64 ± 16	< 0.001
Gender, *n* (%)					< 0.001
Female	451 (36.6)	469 (37.7)	556 (43.7)	590 (47.2)	
Male	782 (63.4)	776 (62.3)	717 (56.3)	660 (52.8)	
Ethnicity, *n* (%)					< 0.001
White	878 (71.2)	886 (71.2)	897 (70.5)	901 (72.1)	
Black	72 (5.8)	78 (6.3)	119 (9.3)	116 (9.3)	
Other	283 (23)	281 (22.5)	257 (20.2)	233 (18.6)	
ICU LOS, day	7.6 ± 8.2	7.3 ± 8.0	8.0 ± 8.9	9.5 ± 10.0	< 0.001
Vasopressin use, *n* (%)	301 (24.4)	261 (21.0)	264 (20.7)	266 (21.3)	0.091
Ventilator use, *n* (%)	502 (40.7)	439 (35.3)	378 (29.7)	330 (26.4)	< 0.001
Comorbidities, *n* (%)					
Atrial fibrillation	365 (29.6)	366 (29.4)	395 (31.0)	438 (35.0)	0.008
Coronary artery disease	392 (31.8)	341 (27.4)	266 (20.9)	202 (16.2)	< 0.001
Congestive heart failure	282 (22.9)	362 (29.1)	444 (34.9)	441 (35.3)	< 0.001
Chronic liver disease	316 (25.6)	259 (20.8)	146 (11.5)	69 (5.5)	< 0.001
Renal disease	148 (12)	167 (13.4)	229 (18.0)	233 (18.6)	< 0.001
Pneumonia	283 (23.0)	337 (27.1)	427 (33.5)	468 (37.4)	< 0.001
COPD	9 (0.7)	20 (1.6)	18 (1.4)	13 (1.0)	0.187
ARDS	308 (25.0)	366 (29.4)	499 (39.2)	613 (49.0)	< 0.001
Stroke	40 (3.2)	61 (4.9)	70 (5.5)	41 (3.3)	0.007
Cancer	153 (12.4)	215 (17.3)	287 (22.5)	322 (25.8)	< 0.001
Diabetes uncomplicated	272 (22.1)	332 (26.7)	365 (28.7)	368 (29.4)	< 0.001
Laboratory parameters					
WBC, 10^9^/L	12.1 ± 12.4	12.8 ± 11.6	13.7 ± 14.5	14.8 ± 10.7	< 0.001
Creatine, mEq/L	1.4 ± 1.3	1.5 ± 1.4	1.9 ± 2.0	2.1 ± 2.1	< 0.001
BUN, mg/dL	25.9 ± 20.5	28.5 ± 22.3	33.7 ± 25.9	39.3 ± 28.5	< 0.001
Anion gap, mmol/L	15.2 ± 5.0	15.6 ± 5.2	15.8 ± 4.8	16.1 ± 5.1	< 0.001
Bicarbonate, mg/dL	22.0 ± 4.8	22.3 ± 4.9	22.1 ± 5.3	21.5 ± 5.6	0.001
Bilirubin, mg/dL	3.9 ± 7.8	2.8 ± 5.7	2.0 ± 4.4	2.0 ± 3.9	< 0.001
Glucose, mg/dL	145.2 ± 70.0	153.7 ± 82.6	155.4 ± 83.5	150.9 ± 87.6	0.010
Hematocrit, %	30.5 ± 7.0	31.9 ± 7.0	32.0 ± 6.7	31.0 ± 6.3	< 0.001
Sodium, mmol/L	138.5 ± 5.2	138.4 ± 7.6	138.5 ± 5.5	138.1 ± 5.9	0.248
Potassium, mmol/L	4.2 ± 0.7	4.2 ± 0.8	4.2 ± 0.9	4.2 ± 0.9	0.844
Platelet, 10^9^/L	151.1 ± 95.3	180.0 ± 107.5	206.0 ± 128.6	241.3 ± 161.5	< 0.001
Chloride, mmol/L	106.0 ± 6.8	105.4 ± 6.8	105.0 ± 6.9	104.8 ± 7.2	< 0.001
Hemoglobin, g/dL	10.5 ± 2.5	10.9 ± 2.4	10.7 ± 2.3	10.3 ± 2.1	< 0.001
PT, seconds	19.2 ± 10.9	17.3 ± 8.3	17.7 ± 11.2	18.5 ± 12.8	< 0.001
APTT, seconds	48.7 ± 28.7	42.4 ± 25.7	39.9 ± 23.2	40.6 ± 24.5	< 0.001
Scoring systems					
SOFA	7.1 ± 3.9	6.5 ± 3.8	6.6 ± 3.7	7.1 ± 4.0	< 0.001
SAPSII	41.3 ± 14.9	41.4 ± 14.5	44.5 ± 15.2	46.7 ± 15.6	< 0.001
AKI stage, *n* (%)					< 0.001
Stage 1	78 (6.3)	88 (7.1)	149 (11.7)	170 (13.6)	
Stage 2	260 (21.1)	277 (22.2)	309 (24.3)	404 (32.3)	
Stage 3	895 (72.6)	880 (70.7)	815 (64.0)	676 (54.1)	
RRT, *n* (%)	54 (4.4)	82 (6.6)	101 (7.9)	60 (4.8)	< 0.001
In hospital mortality, *n* (%)	256 (20.8)	257 (20.6)	369 (29.0)	389 (31.1)	< 0.001

*ARDS* acute respiratory distress syndrome; *COPD* Chronic obstructive pulmonary disease; *BUN* blood urea nitrogen; *WBC* white blood cell; *PT* prothrombin time; *APTT* activated partial thromboplastin time; *SOFA* sequential organ failure assessment; *SAPSII* simplified acute physiology score II; *AKI* acute kidney injury; *ICU* intensive care unit; *LOS* length of stay; RRT renal replacement therapy

### FAR level and in-hospital mortality

**Fig. 1:**
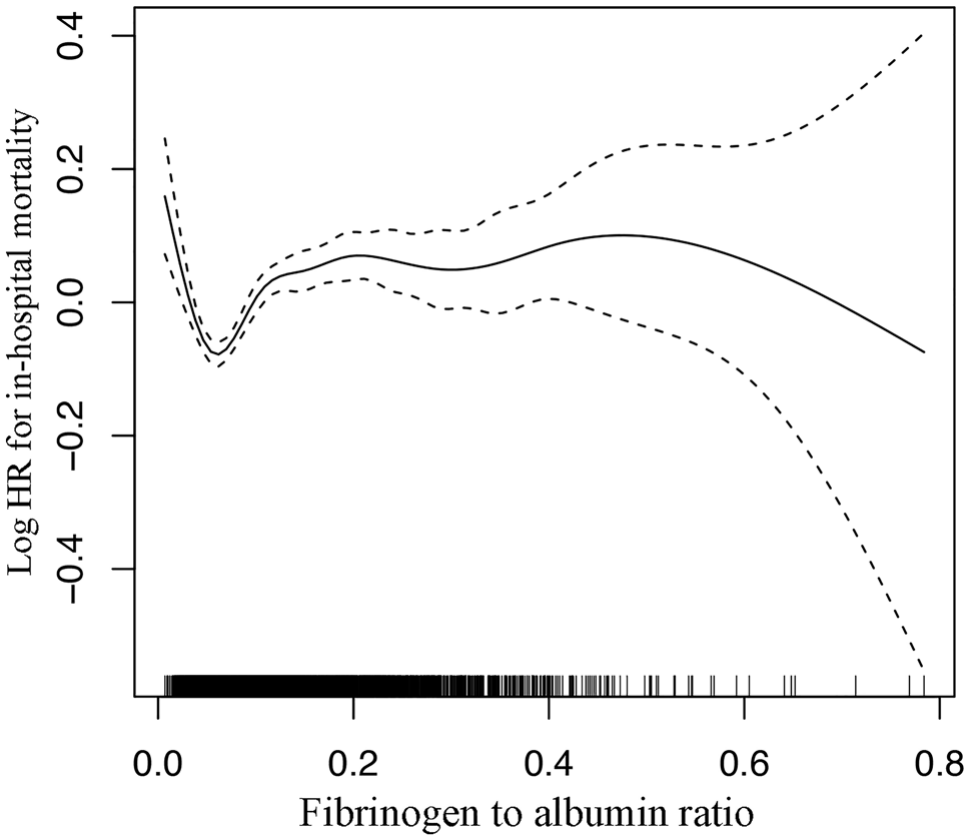
Association between FAR and in-hospital mortality. A threshold, nonlinear association between FAR and in-hospital mortality was observed in a generalized additive model

The relationship between FAR level and in-hospital mortality was non-liner, and a U-shaped curve was observed (Fig. 1). We used multivariate Cox regression analysis to determine the association between FAR and in-hospital mortality in critically ill patients with AKI (Table 2). Following the stratification of FAR into quartiles and using the second quartile as reference group. In model I, the top quartile of FAR (FAR ≥ 0.157) was associated with increased risk of in-hospital mortality after adjustment for age, gender and ethnicity (HR, 95% CI 1.57, 1.33–1.84). In model II, after adjustment for age, gender, ethnicity, vasopressin use, ventilator use, atrial fibrillation, coronary artery disease, congestive heart failure, chronic liver disease, chronic kidney disease, stroke, malignancy, pneumonia, ARDS, and COPD, FAR was also an independent predictor of in-hospital mortality (HR, 95% CI 1.20, 1.01–1.42). In model III, after adjustment for more confounding factors, higher FAR remained a significant predictor of in-hospital mortality in critically ill patients with AKI (HR, 95% CI 1.48, 1.04–2.10).

**Table 2:** Relationship between quartile of FAR and in-hospital mortality

	Quartile1 (*N* = 1233)		Quartile3 (*N* = 1273)		Quartile4 (*N* = 1250)	
	HR (95%CI)	*P* value	HR (95%CI)	*P* value	HR (95%CI)	*P* value
Unadjusted	1.02 (0.85, 1.21)	0.841	1.36 (1.16, 1.59)	< 0.001	1.54 (1.32, 1.81)	< 0.001
Model I	1.02 (0.86, 1.21)	0.813	1.37 (1.17, 1.61)	< 0.001	1.57 (1.33, 1.84)	< 0.001
Model II	0.91 (0.77, 1.09)	0.308	1.10 (0.93, 1.30)	0.250	1.20 (1.01, 1.42)	0.037
Model III	1.04 (0.86, 1.27)	0.692	1.24 (0.98, 1.56)	0.068	1.48 (1.04, 2.10)	0.028

Reference group was Quartile 2

*HR* hazard ratio

Model I: adjusted for age, gender and ethnicity

Model II: adjusted for model I covariates and vasopressin use, ventilator use, atrial fibrillation, coronary artery disease, congestive heart failure, chronic liver disease, renal disease, stroke, cancer, pneumonia, acute respiratory distress syndrome, chronic obstructive pulmonary disease and RRT

Model III: adjusted for model II covariates and hemoglobin, WBC, creatine, BUN, anion gap, bicarbonate, bilirubin, glucose, hematocrit, sodium, potassium, platelet, chloride, hemoglobin, PT, APTT, AKI stage

### Subgroup analysis

We performed subgroup analysis to assess the association between FAR and in-hospital mortality (Table 3). There was no statistically significant in several strata (*P* for interaction > 0.05). We found that patients with higher FAR had significantly higher mortality with SOFA score < 5 and SAPSII score < 39. Similar trends were observed in patients with congestive heart failure, coronary artery disease, atrial fibrillation, pneumonia and cancer.

**Table 3:** Subgroup analysis of the association between FAR and in-hospital mortality

Characteristic	*N*	HR (95%CI)	*P* value	*P* for interaction
Age				0.227
< 65	2571	1.64 (1.32, 2.04)	< 0.001	
≥ 65	2430	1.82 (1.45, 2.29)	< 0.001	
Gender				0.506
Female	2066	1.52 (1.19, 1.94)	0.001	
Male	2935	1.86 (1.51, 2.29)	< 0.001	
Vasopressin use				< 0.001
No	3909	1.11 (0.90, 1.38)	0.327	
Yes	1092	1.63 (1.23, 2.17)	0.001	
Ventilator use				0.403
No	3352	1.50 (1.24, 1.81)	< 0.001	
Yes	1649	1.51 (1.15, 1.97)	0.003	
AFIB				0.009
No	3437	1.25 (1.03, 1.51)	0.025	
Yes	1564	1.58 (1.11, 2.24)	0.010	
CAD				< 0.001
No	3800	1.27 (1.07, 1.50)	0.005	
Yes	1201	6.01 (3.70, 9.74)	< 0.001	
CHF				0.001
No	3472	1.62 (1.33, 1.95)	< 0.001	
Yes	1529	1.86 (1.38, 2.50)	< 0.001	
CLD				< 0.001
No	4211	2.28 (1.88, 2.77)	< 0.001	
Yes	790	1.19 (0.78, 1.79)	0.421	
CKD				0.022
No	4224	1.76 (1.48, 2.09)	< 0.001	
Yes	777	1.35 (0.92, 1.99)	0.128	
COPD				0.502
No	4941	1.73 (1.47, 2.02)	< 0.001	
Yes	60	0.86 (0.17, 4.26)	0.852	
ARDS				< 0.001
No	3215	2.09 (1.63, 2.68)	< 0.001	
Yes	1786	0.98 (0.79, 1.19)	0.751	
Pneumonia				< 0.001
No	3486	1.40 (1.15, 1.71)	0.001	
Yes	1515	1.49 (1.14, 1.95)	0.003	
Stroke				0.002
No	4789	1.74 (1.48, 2.05)	< 0.001	
Yes	212	1.09 (0.53, 2.23)	0.823	
Cancer				< 0.001
No	4024	1.55 (1.29, 1.86)	< 0.001	
Yes	977	1.86 (1.30, 2.65)	0.001	
Diabetes				0.671
No	3664	1.61 (1.34, 1.93)	< 0.001	
Yes	1337	2.02 (1.45, 2.80)	< 0.001	
RRT				< 0.001
No	4704	1.02 (0.84, 1.23)	0.876	
Yes	297	1.73 (1.23, 2.44)	0.002	
AKI stage				< 0.001
Stage 1	485	0.67(0.43, 1.03)	0.065	
Stage 2	1250	1.20 (0.92, 1.56)	0.184	
Stage 3	3266	2.10 (1.69, 2.63)	< 0.001	
WBC, 10^9^/L				0.032
< 11.2	2479	1.06 (0.85, 1.33)	0.600	
≥ 11.2	2522	1.28 (1.01, 1.61)	0.039	
Creatine, mEq/L				< 0.001
< 1.1	2173	1.17 (0.89, 1.54)	0.273	
≥ 1.1	2828	1.11 (0.91, 1.34)	0.306	
BUN, mg/dL				< 0.001
< 23	2382	1.22 (0.90, 1.65)	0.207	
≥ 23	2619	0.95 (0.79, 1.14)	0.576	
Anion gap, mmol/L				< 0.001
< 15	2310	1.32 (1.00, 1.74)	0.052	
≥ 15	2679	1.07 (0.89, 1.30)	0.470	
Bicarbonate, mg/dL				< 0.001
< 22	2192	0.95 (0.78, 1.16)	0.604	
≥ 22	2809	1.48 (1.15, 1.92)	0.003	
Bilirubin, mg/dL				< 0.001
< 0.8	2089	2.53 (1.74, 3.66)	< 0.001	
≥ 0.8	2567	1.01 (0.83, 1.22)	0.915	
Glucose, mg/dL				0.301
< 133	2447	1.16 (0.92, 1.45)	0.206	
≥ 133	2538	1.18 (0.95, 1.48)	0.137	
Hematocrit, %				0.574
< 30.8	2496	1.08 (0.88, 1.35)	0.456	
≥ 30.8	2505	1.29 (1.02, 1.64)	0.035	
Sodium, mmol/L				0.006
< 139	2381	1.00 (0.80, 1.25)	0.977	
≥ 139	2618	1.38 (1.11, 1.73)	0.005	
Potassium, mmol/L				< 0.001
< 4.1	2225	1.05 (0.82, 1.34)	0.714	
≥ 4.1	2767	1.30 (1.06, 1.60)	0.011	
Platelet, 10^9^/L				< 0.001
< 168	2487	1.20 (0.98, 1.47)	0.071	
≥ 168	2514	1.61 (1.19, 2.18)	0.002	
Chloride, mmol/L				< 0.001
< 105	2214	1.05 (0.84, 1.32)	0.658	
≥ 105	2787	1.28 (1.02, 1.60)	0.032	
Hemoglobin, g/dL				0.008
< 10.4	2480	1.12 (0.91, 1.38)	0.276	
≥ 10.4	2521	1.26 (0.99, 1.61)	0.061	
PT, seconds				< 0.001
< 15.5	2451	1.57 (1.15, 2.13)	0.005	
≥ 15.5	2547	1.20 (0.99, 1.45)	0.067	
APTT, seconds				< 0.001
< 35	2488	1.44 (1.06, 1.96)	0.021	
≥ 35	2509	1.27 (1.05, 1.54)	0.016	
SOFA score				< 0.001
< 5	1542	2.90 (1.85, 4.86)	< 0.001	
≥ 5	3459	1.58 (1.33, 1.87)	< 0.001	
SAPSII score				< 0.001
< 39	2020	1.61 (1.10, 2.36)	0.014	
≥ 39	2981	1.45 (1.22, 1.73)	< 0.001	

*ARDS* acute respiratory distress syndrome, *COPD* chronic obstructive pulmonary disease, *BUN* blood urea nitrogen, *WBC* white blood cell, *PT* prothrombin time, *APTT* activated partial thromboplastin time, *SOFA* sequential organ failure assessment, *SAPSII* simplified acute physiology score II

### Prediction of mortality

**Fig. 2:**
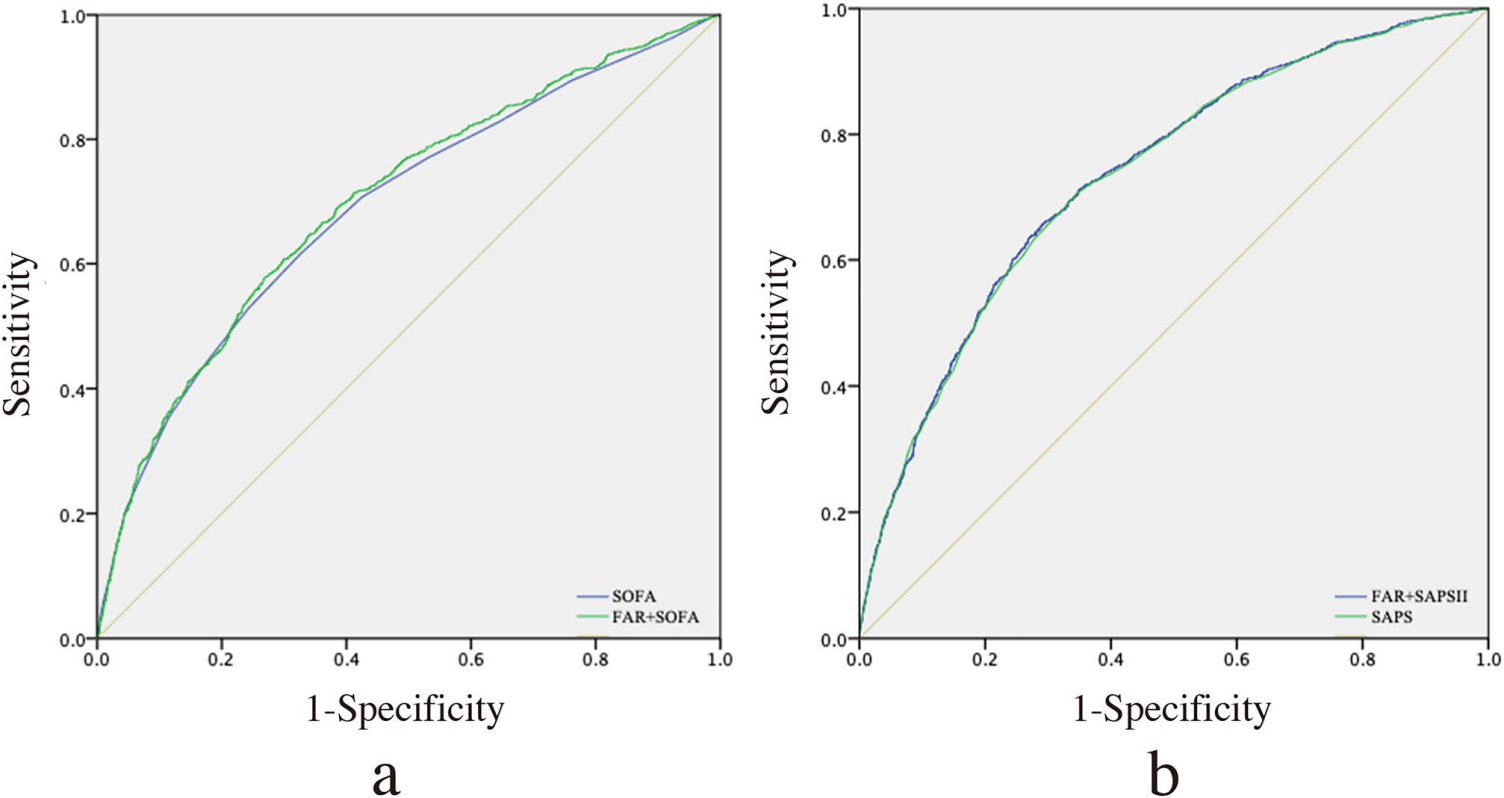
ROC curves for the prediction of in-hospital mortality. **a** The ability of SOFA scores and FAR plus SOFA scores to predict in-hospital mortality. **b** The ability of SAPSII scores and FAR plus SAPSII scores to predict in-hospital mortality

The ROC curves were generated using the indicated variables (Fig. 2). The AUC for SOFA score was 0.690, compared to 0.700 for FAR plus SOFA score (*P* < 0.001). Moreover, the AUCs for SAPSII score and FAR plus SAPSII score were 0.734 and 0.736, respectively (*P* < 0.001).

## Discussion

In our study, we observed an elevated FAR level was associated with increased risk of in-hospital mortality after adjusting for important confounding factors, and the relationship between FAR and mortality was nonlinear. To the best of our knowledge, this is the first study to measure the association between FAR and in-hospital mortality in critically ill patients with AKI.

The FAR is a combination of fibrinogen and albumin level that serves as a potential prognostic biomarker to predict risk for various diseases. Zou et al. reported the association between the FAR level and mortality in peritoneal dialysis (PD) patients, and they found that FAR was an independent predictor of both all-cause and cardiovascular disease (CVD) mortality [19]. Liu et al. suggested that a high FAR strongly correlated with worse overall survival in renal cancer patients [20]. Another retrospective study explored a significant association between higher FAR and occurrence of AKI in patients undergoing percutaneous coronary intervention [15].

Currently, fibrinogen has been shown to go beyond its traditional role in coagulation and to recognized as an acute-phase response protein [21]. It has been proposed that FAR can be utilized as a readily available indicator for assessing microinflammation [10, 12], and recent studies have associated it with inflammation-based disease, which is associated with increased mortality in critically ill patients [22]. Similarly, the present study showed that FAR was an independent predictor in critically patients with AKI. The following reason might be accounted for in our findings. Many immune cells involved in acute and chronic inflammation generated and released a wide variety of inflammatory factors, such as transforming growth factor-β (TGF-β), vascular endothelial growth factor (VEGF), and platelet-derived growth factor (PDGF) [23] [24]. However, the precise mechanism needs further investigation. Elevated fibrinogen level resulted in activation of proinflammatory cascades, which lead to the release of inflammatory cytokines and thereby contribute to the occurrence and development of inflammation diseases [25, 26]. Moreover, inhibition of fibrinogen resulted in reduced inflammation and an attenuated disease course [27]. Previous studies have reported that plasma fibrinogen level in AKI patients was higher than the normal range and an increased blood viscosity due to elevated fibrinogen level was independently associated with increased risk of cardiovascular events [28–30]. In addition, higher fibrinogen was associated with the presence of AKI in abdominal aortic aneurysm repair patients [29], and predicted the development of AKI in contrast-induced nephropathy [31].

Serum albumin is commonly used to evaluate nutritional status of patients. Recent studies found hypoalbuminemia negatively correlated with patient prognosis was more greatly attributed to systemic inflammation than malnutrition in various disease [32, 33]. Moreover, a nonlinear relationship between albumin and mortality was also observed [34]. A sufficient amount of albumin is likely to protect kidneys from toxic substances and maintain colloid pressure to guarantee perfusion [35], all of which are risk factors for the development of AKI. As reported previously, AKI was associated with local and systemic inflammation [36], as markers of inflammation, fibrinogen and albumin have been investigated as significant predictors in AKI patients [37, 38]. All of these results led us to conclude the predictive value of FAR is enhanced in the AKI population. Next, we further investigated the association between FAR and mortality using a generalized additive model and discovered that the relationship between FAR and in-hospital mortality was nonlinear, exhibiting approximate U-shaped curves. Combined with the basic characteristics results, where patients with roughly normal FAR level (Q2 group) had the lowest mortality rate, suggesting that the FAR level was associated with in-hospital mortality in a U-shaped relationship, indicating that there might be a normal value range of FAR level in the middle of the entire distribution rather than on either side. Extremely low FAR level should also lead to poor prognosis among critically ill patients with AKI. Indeed, a retrospective study in CKD patients demonstrated that lower fibrinogen can lead to an increased risk of bleeding and correlate with increased CV event mortality [39]. However, we did not find that lower FAR was associated with in-hospital mortality in AKI patients. This may be because bleeding is multifactorial and partially due to uremic disturbance of platelet adhesion, anemia, fibrinolysis, and coagulation [40]. The interactive influence among multiple factors could conceal the effect of FAR level alone on the prognosis of critically ill patients with AKI.

The subgroup analysis of in-hospital mortality revealed a positive association of an increased FAR with mortality in AKI patients with CAD. Prior studies have illustrated that cardiovascular disease may affect the outcome of AKI [41]. Interestingly, we found that AKI patients with malignancy were associated with higher mortality. Cancer patients requiring admission to the ICU are typically older, more likely required mechanical ventilation, more susceptible to severe sepsis and a higher incidence of nephrotoxicity induced by targeted therapy, all of which are common risk factors associated with increased ICU readmission or mortality [42, 43]. It is well known that the mortality in cancer patients with AKI was higher than that without AKI [44]. However, a previous study demonstrated that cancer patients with AKI correlated with reduced mortality, indicating a complex and even paradoxical relationship of AKI and clinical risk in cancer patients [45]. Furthermore, we found the risk of AKI was high following pneumonia, this might be related to an increased immune response of pneumonia [46, 47]. Clearly, further research into mechanisms underlying the interaction between potential factors and poor outcomes is required.

There are some unavoidable limitations in our study. First, this was a retrospective study based on a single-center database, and selection bias was inevitable. Second, we only measured FAR upon admission to the ICU, whether infusion of fibrinogen or albumin during ICU stay may affect outcome, especially in patients with high baseline levels should be further verified. Third, we did not adjust all factors related to mortality owing to lack of related data. Finally, we only investigate in-hospital mortality which may affect assessment of prognosis, the follow-up length of mortality should be considered in the future analysis.

## Conclusion

We found a U-shaped relationship between the FAR and mortality and a higher level of FAR was associated with increased risk of in-hospital mortality in critically ill patients with AKI. However, our findings still need to be confirmed by large prospective studies with long follow-up.

## Data Availability

The clinical data used to support the findings of this study were supplied by Monitoring in Intensive Care Database III version 1.4 (MIMIC-III v.1.4). Although the database is publicly and freely available, researchers must complete the National Institutes of Health’s web-based course known as Protecting Human Research Participants to apply for permission to access the database. The datasets used and/or analyzed during the current study are available from the corresponding author on reasonable request.
